# 2-Iodo-4,6-dimethyl­pyrimidine

**DOI:** 10.1107/S1600536810005660

**Published:** 2010-02-24

**Authors:** Qing-Min Jiang, Gui-Yun Mao, Lu-Ye Hao, Xin-Qi Hao, Mao-Ping Song

**Affiliations:** a Henan Industrial University Chemical Technology Vocational College, Zhengzhou 450042, People’s Republic of China; b Department of Chemistry, Henan Key Laboratory of Chemical, Biology and Organic Chemistry, Zhengzhou University, Zhengzhou 450052, People’s Republic of China

## Abstract

In the title compound, C_6_H_7_IN_2_, the non-H atoms of the mol­ecule are located on a crystallographic mirror plane; the H atoms of the methyl groups are therefore disordered over two positions of equal occupancy. In the crystal structure, short inter­molecular I⋯N contacts [3.390 (3) Å] are found, linking the mol­ecules into zigzag chains. In addition, there are inter­molecular π–π stacking inter­actions between the pyrimidine rings of adjacent mol­ecules [centroid–centroid distance = 3.5168 (10) Å], resulting in a two-dimensional supra­molecular architecture.

## Related literature

For applications of pyrimidine derivatives, see: Chinchilla *et al.* (2004 ▶); Xu *et al.* (2009*a* ▶,*b* ▶). For halogen–electronegative atom inter­actions, see: Lommerse *et al.* (1996 ▶). For the synthesis of 4,6-dimethyl-2-chloro­pyrimidine, see: Kosolapoff & Roy (1961 ▶) and literature cited therein.
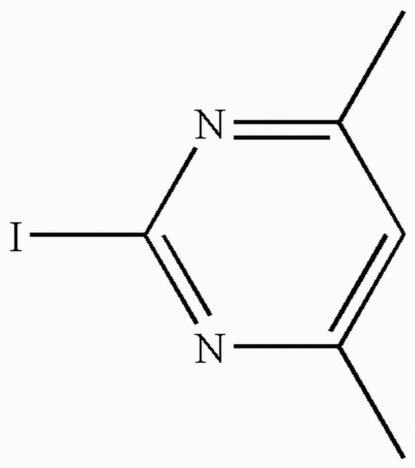


## Experimental

### 

#### Crystal data

C_6_H_7_IN_2_
*M* *_r_* = 234.04
Orthorhombic, 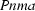

*a* = 7.930 (2) Å
*b* = 7.0256 (19) Å
*c* = 14.499 (4) Å
*V* = 807.8 (4) Å^3^
*Z* = 4
Mo *K*α radiation
μ = 3.88 mm^−1^
*T* = 296 K
0.32 × 0.25 × 0.21 mm

#### Data collection

Bruker SMART APEXII CCD area-detector diffractometer
Absorption correction: multi-scan (*SADABS*; Sheldrick, 1996 ▶) *T*_min_ = 0.370, *T*_max_ = 0.496
5541 measured reflections
817 independent reflections
739 reflections with *I* > 2σ(*I*)
*R*_int_ = 0.029

#### Refinement

*R*[*F* ^2^ > 2σ(*F* ^2^)] = 0.026
*wR*(*F* ^2^) = 0.066
*S* = 1.10
817 reflections
57 parameters
H-atom parameters constrained
Δρ_max_ = 0.81 e Å^−3^
Δρ_min_ = −0.21 e Å^−3^

### 

Data collection: *APEX2* (Bruker, 2004 ▶); cell refinement: *SAINT* (Bruker, 2004 ▶); data reduction: *SAINT*; program(s) used to solve structure: *SHELXS97* (Sheldrick, 2008 ▶); program(s) used to refine structure: *SHELXL97* (Sheldrick, 2008 ▶); molecular graphics: *SHELXTL* (Sheldrick, 2008 ▶); software used to prepare material for publication: *SHELXL97*.

## Supplementary Material

Crystal structure: contains datablocks global, I. DOI: 10.1107/S1600536810005660/si2242sup1.cif

Structure factors: contains datablocks I. DOI: 10.1107/S1600536810005660/si2242Isup2.hkl

Additional supplementary materials:  crystallographic information; 3D view; checkCIF report

## Figures and Tables

**Fig. 1.**
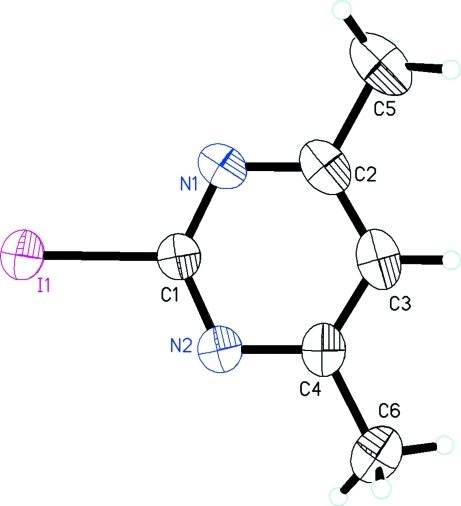
The molecular structure of the title compound with displacement ellipsoids at the 30% probability level, the disordered H atoms are omitted.

**Fig. 2.**
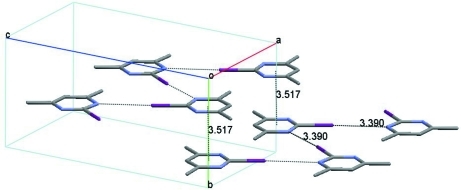
Partial view of the crystal packing showing the short intermolecular I···N contacts and π—π stacking interactions.

## supplementary crystallographic information

### Comment

Some derivatives of pyrimidine are important chemical materials (Chinchilla *et al.*, 2004). Among them, 4,6-dimethyl-2-iodopyrimidine is a good partner in cross-coupling reaction giving a variety of pyrimidine ligands (Xu *et al.*, 2009a, b). The molecular structure of the related title compound is shown in Fig. 1. The molecule is located on a crystallographic mirror plane, thus the H atoms of the methyl groups are disordered over two positions, with site-occupation factors fixed at 0.5. The interesting feature of the crystal structure is short intermolecular I···N contacts [3.390 (3) Å] (Lommerse *et al.*, 1996), which is obviously shorter than the sum of the van der Waals radii of the relevant atoms. In addtion, there are strong intermolecular π—π stacking interactions between the pyrimidine rings of adjacent molecules [centroid-centroid distance = 3.5168 (10) Å], resulting in a two-dimensional supramolecular architecture (Fig.2).

### Experimental

The title compound was prepared as described in literature (Kosolapoff & Roy 1961) and recrystallized from dichloromethane-petroleum ether solution at room temperature to give the desired product as colourless crystals suitable for single-crystal X-ray diffraction.

### Refinement

H atoms attached to C atoms of the title compound were placed in geometrically idealized positions and treated as riding with C—H distances constrained to 0.93–0.96 Å, and with *U*_iso_(H) = 1.2*U*_eq_(C) and (1.5*U*_eq_ for methyl H).

### Crystal data

**Table:**

C_6_H_7_IN_2_	*D*_x_ = 1.924 Mg m^−^^3^
*M**_r_* = 234.04	Mo *K*α radiation, λ = 0.71073 Å
Orthorhombic, *P**n**m**a*	Cell parameters from 2976 reflections
*a* = 7.930 (2) Å	θ = 2.8–24.9°
*b* = 7.0256 (19) Å	µ = 3.88 mm^−^^1^
*c* = 14.499 (4) Å	*T* = 296 K
*V* = 807.8 (4) Å^3^	Block, colourless
*Z* = 4	0.32 × 0.25 × 0.21 mm
*F*(000) = 440	

### Data collection

**Table:**

Bruker SMART APEXII CCD area-detector diffractometer	817 independent reflections
Radiation source: fine-focus sealed tube	739 reflections with *I* > 2σ(*I*)
graphite	*R*_int_ = 0.029
phi and ω scans	θ_max_ = 25.5°, θ_min_ = 2.8°
Absorption correction: multi-scan (*SADABS*; Sheldrick, 1996)	*h* = −9→9
*T*_min_ = 0.370, *T*_max_ = 0.496	*k* = −8→8
5541 measured reflections	*l* = −17→17

### Refinement

**Table:**

Refinement on *F*^2^	Primary atom site location: structure-invariant direct methods
Least-squares matrix: full	Secondary atom site location: difference Fourier map
*R*[*F*^2^ > 2σ(*F*^2^)] = 0.026	Hydrogen site location: inferred from neighbouring sites
*wR*(*F*^2^) = 0.066	H-atom parameters constrained
*S* = 1.10	*w* = 1/[σ^2^(*F*_o_^2^) + (0.0375*P*)^2^ + 0.259*P*] where *P* = (*F*_o_^2^ + 2*F*_c_^2^)/3
817 reflections	(Δ/σ)_max_ = 0.001
57 parameters	Δρ_max_ = 0.81 e Å^−^^3^
0 restraints	Δρ_min_ = −0.21 e Å^−^^3^

### Special details

**Table:**

Geometry. All esds (except the esd in the dihedral angle between two l.s. planes)are estimated using the full covariance matrix. The cell esds are takeninto account individually in the estimation of esds in distances, anglesand torsion angles; correlations between esds in cell parameters are onlyused when they are defined by crystal symmetry. An approximate (isotropic)treatment of cell esds is used for estimating esds involving l.s. planes.
Refinement. Refinement of *F*^2^ against ALL reflections. The weighted *R*-factor wR andgoodness of fit *S* are based on *F*^2^, conventional *R*-factors *R* are basedon *F*, with *F* set to zero for negative *F*^2^. The threshold expression of*F*^2^ > σ(*F*^2^) is used only for calculating *R*-factors(gt) etc. and isnot relevant to the choice of reflections for refinement. *R*-factors basedon *F*^2^ are statistically about twice as large as those based on *F*, and *R*-factors based on ALL data will be even larger.

### Fractional atomic coordinates and isotropic or equivalent isotropic displacement parameters (Å^2^)

**Table:**

	*x*	*y*	*z*	*U*_iso_*/*U*_eq_	Occ. (<1)
C1	0.4205 (6)	0.2500	0.4216 (3)	0.0559 (10)	
C2	0.4224 (8)	0.2500	0.5763 (3)	0.0708 (13)	
C3	0.5963 (8)	0.2500	0.5741 (3)	0.0746 (14)	
H3	0.6587	0.2500	0.6285	0.090*	
C4	0.6751 (7)	0.2500	0.4899 (3)	0.0680 (12)	
C5	0.3278 (11)	0.2500	0.6665 (5)	0.108 (2)	
H5A	0.2299	0.1703	0.6612	0.162*	0.50
H5B	0.2935	0.3775	0.6812	0.162*	0.50
H5C	0.3996	0.2022	0.7145	0.162*	0.50
C6	0.8651 (8)	0.2500	0.4820 (5)	0.107 (2)	
H6A	0.9047	0.1215	0.4761	0.161*	0.50
H6B	0.9132	0.3068	0.5361	0.161*	0.50
H6C	0.8980	0.3218	0.4286	0.161*	0.50
I1	0.27885 (5)	0.2500	0.29859 (2)	0.07396 (18)	
N1	0.3314 (6)	0.2500	0.4980 (2)	0.0656 (9)	
N2	0.5862 (5)	0.2500	0.4101 (2)	0.0616 (9)	

### Atomic displacement parameters (Å^2^)

**Table:**

	*U*^11^	*U*^22^	*U*^33^	*U*^12^	*U*^13^	*U*^23^
C1	0.063 (3)	0.046 (2)	0.058 (2)	0.000	−0.002 (2)	0.000
C2	0.107 (4)	0.047 (2)	0.058 (3)	0.000	0.007 (3)	0.000
C3	0.106 (4)	0.059 (3)	0.059 (3)	0.000	−0.014 (3)	0.000
C4	0.076 (3)	0.064 (3)	0.064 (3)	0.000	−0.012 (2)	0.000
C5	0.158 (7)	0.098 (4)	0.068 (3)	0.000	0.022 (4)	0.000
C6	0.073 (4)	0.151 (6)	0.098 (4)	0.000	−0.014 (3)	0.000
I1	0.0685 (3)	0.0874 (3)	0.0660 (3)	0.000	−0.01043 (13)	0.000
N1	0.075 (2)	0.065 (2)	0.057 (2)	0.000	0.0106 (19)	0.000
N2	0.064 (2)	0.062 (2)	0.059 (2)	0.000	−0.0017 (17)	0.000

### Geometric parameters (Å, °)

**Table:**

C1—N1	1.314 (6)	C4—N2	1.356 (6)
C1—N2	1.325 (6)	C4—C6	1.511 (9)
C1—I1	2.108 (4)	C5—H5A	0.9600
C2—N1	1.345 (7)	C5—H5B	0.9600
C2—C3	1.380 (9)	C5—H5C	0.9600
C2—C5	1.507 (8)	C6—H6A	0.9600
C3—C4	1.371 (7)	C6—H6B	0.9600
C3—H3	0.9300	C6—H6C	0.9600
			
N1—C1—N2	129.8 (4)	C2—C5—H5B	109.5
N1—C1—I1	115.3 (3)	H5A—C5—H5B	109.5
N2—C1—I1	114.9 (3)	C2—C5—H5C	109.5
N1—C2—C3	121.1 (5)	H5A—C5—H5C	109.5
N1—C2—C5	117.7 (6)	H5B—C5—H5C	109.5
C3—C2—C5	121.2 (6)	C4—C6—H6A	109.5
C4—C3—C2	118.4 (5)	C4—C6—H6B	109.5
C4—C3—H3	120.8	H6A—C6—H6B	109.5
C2—C3—H3	120.8	C4—C6—H6C	109.5
N2—C4—C3	121.6 (5)	H6A—C6—H6C	109.5
N2—C4—C6	116.9 (4)	H6B—C6—H6C	109.5
C3—C4—C6	121.5 (5)	C1—N1—C2	115.1 (5)
C2—C5—H5A	109.5	C1—N2—C4	114.1 (4)
			
N1—C2—C3—C4	0.0	C3—C2—N1—C1	0.0
C5—C2—C3—C4	180.0	C5—C2—N1—C1	180.0
C2—C3—C4—N2	0.0	N1—C1—N2—C4	0.0
C2—C3—C4—C6	180.0	I1—C1—N2—C4	180.0
N2—C1—N1—C2	0.0	C3—C4—N2—C1	0.00
I1—C1—N1—C2	180.0	C6—C4—N2—C1	180.0
